# A questionnaire measuring staff perceptions of Lean adoption in healthcare: development and psychometric testing

**DOI:** 10.1186/s12913-017-2163-x

**Published:** 2017-03-24

**Authors:** Monica Kaltenbrunner, Lars Bengtsson, Svend Erik Mathiassen, Maria Engström

**Affiliations:** 1 Faculty of Health and Occupational Studies, University of Gävle, 801 76 Gävle, Sweden; 2 Faculty of Engineering and Sustainable Development, University of Gävle, 801 76 Gävle, Sweden; 3 Department of Public Health and Caring Sciences, Uppsala University, Uppsala, Sweden; 4 Nursing Department, Medicine and Health College, Lishui University, Lishui Shi, China

**Keywords:** Service sector, Healthcare sector, Lean maturity, Instrument development, Survey instrument, Assessment instrument, Performance measurement, Questionnaire validity, Validity, Reliability

## Abstract

**Background:**

During the past decade, the concept of Lean has spread rapidly within the healthcare sector, but there is a lack of instruments that can measure staff’s perceptions of Lean adoption. Thus, the aim of the present study was to develop a questionnaire measuring Lean in healthcare, based on Liker’s description of Lean, by adapting an existing instrument developed for the service sector.

**Methods:**

A mixed-method design was used. Initially, items from the service sector instrument were categorized according to Liker’s 14 principles describing Lean within four domains: *philosophy*, *processes*, *people and partners* and *problem*-*solving*. Items were lacking for three of Liker’s principles and were therefore developed de novo. Think-aloud interviews were conducted with 12 healthcare staff from different professions to contextualize and examine the face validity of the questionnaire prototype. Thereafter, the adjusted questionnaire’s psychometric properties were assessed on the basis of a cross-sectional survey among 386 staff working in primary care.

**Results:**

The think-aloud interviews led to adjustments in the questionnaire to better suit a healthcare context, and the number of items was reduced. Confirmatory factor analysis of the adjusted questionnaire showed a generally acceptable correspondence with Liker’s description of Lean. Internal consistency, measured using Cronbach’s alpha, for the factors in Liker’s description of Lean was 0.60 for the factor *people and partners*, and over 0.70 for the three other factors. Test-retest reliability measured by the intra-class correlation coefficient ranged from 0.77 to 0.88 for the four factors.

**Conclusions:**

We designed a questionnaire capturing staff’s perceptions of Lean adoption in healthcare on the basis of Liker’s description. This Lean in Healthcare Questionnaire (LiHcQ) showed generally acceptable psychometric properties, which supports its usability for measuring Lean adoption in healthcare. We suggest that further research focus on verifying the usability of LiHcQ in other healthcare settings, and on adjusting the instrument if needed.

**Electronic supplementary material:**

The online version of this article (doi:10.1186/s12913-017-2163-x) contains supplementary material, which is available to authorized users.

## Background

During the past decade, interest in adopting Lean in the healthcare sector has increased [1], the primary aims of implementation being to improve the quality of care [2] and to increase efficiency [3]. Adopting Lean and let it become a natural part of daily work routines is challenging [1, 4]. Most common is to adopt Lean to some extent and limited to certain parts of the organization [1, 5–8]. In such cases, system-wide improvements cannot be expected [9]. A recent review [10] of Lean in healthcare concluded that research is needed on how to evaluate the extent of Lean adoption and on how Lean is perceived by healthcare staff. Thus, the aim of the present study was to develop a questionnaire measuring staff perception of Lean adoption in healthcare, including an analysis of its psychometric properties.

### Liker’s description of Lean and Lean in healthcare

**Fig. 1:**
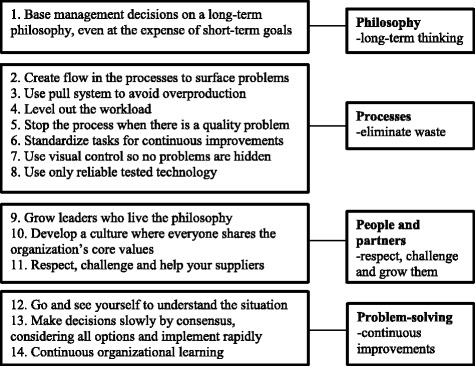
Lean as described by Liker [14] in terms of 4 domains and 14 principles

One challenge when describing Lean adoption is that there is no consensus concerning how to define Lean, and the principles of Lean can be expressed and understood in several different ways [1, 11–13]. In the present study, we have chosen Liker’s [14] description of Lean. Other descriptions have been proposed by, for instance, Womack, Jones and Roos [15], whose description of Lean is similar to Liker’s, cited frequently and described extensively. However, their description has been criticized for not paying attention to the human resources in a Lean organization [16]. Another framing of Lean was suggested by Shah and Ward [17]. Their description of Lean, however, lacks a long-term perspective and does not address decentralized decision-making, which is important in healthcare. Spear and Bowen [18] also described Lean adapted in the industry sector, using four core aspects. Liker’s [14] description of Lean was considered best suited for this study as the principles included are quite generic, include both an operative and a philosophical side of Lean, and stress human resources [14]. Liker identifies 14 central principles in four domains: *philosophy*, *processes*, *people and partners* and *problem*-*solving* (the 4P) (Fig. 1). According to Liker [14], the domain *philosophy* means basing decisions on long-term thinking aiming to creating values both for the individual patient and for society as a whole, with the customer in focus, which is something the entire organization should strive for. Similarities with healthcare are the focus on the customer and on creating values for the patient [19]. Further, Liker [14] described the domain *processes*, which address initiatives to increase quality and efficiency, mainly by using the allocated resources optimally and reducing waste. This can be achieved by mapping processes and improving flow. When flow is optimal, there is no or minimal waste, the staff know what is expected of them, when to do what and they also know what their colleagues are doing and can see the importance of each part of the whole process. Reducing waste means reducing what does not add value to the product or service, from the customer/patient perspective. Waste includes waiting time, unnecessary movements, product defects and not using employees’ creativity. The domain *people and partners* involves respecting and challenging people and enabling them to grow, within and in connection with the organization [14]. Respecting people and enabling their growth are also central in healthcare as the care provided should be person-centered, respect and enabling should also apply to staff, partners and suppliers [19–21]. This approach also includes the organization’s responsibility for enabling staff and giving them the prerequisites to provide high-quality patient care [19]. The domain *problem*-*solving* aims at achieving the right quality and flow in the organization by finding the root causes of problems. Staff members continuously solve problems and are, in this way, involved in evaluations, decisions and development of their workplace. Thus, we found Liker’s [14] description to be the most useful when developing a questionnaire measuring Lean in healthcare.

### Instruments measuring Lean

Different instruments have been developed to measure Lean in different occupational sectors (e.g., [17, 22–29]); they are based on different conceptual foundations, entail different data collection methods, use different respondents and are mostly developed for the industry sector. According to Guillemin et al. [30], when selecting an instrument it is important that it suits the context it is to be used in. Hence, we did not consider instruments developed for industry as a basis for further development into an instrument suitable in a healthcare context. It is reasonable that an instrument intended to measure Lean in healthcare should include the core values of the healthcare professions, i.e. to enable people and show respect for them, as is done in person-centered care [19]. Another important aspect is to adapt the instrument to those who can provide the requested information [31], in this case the staff. We found two instruments from sectors other than industry that we regarded as interesting candidates for further development in the present study: Roszell’s [29] and Malmbrandt and Åhlström’s [28] instruments. Roszell’s [29] instrument is specifically developed for healthcare. The questionnaire is based on expert opinions and literature describing Lean, and the intended respondents are nurses. However, it consists of 110 items, which we consider to be an unfeasible size for regular use by practitioners. Malmbrandt and Åhlström [28] developed their instrument in European service sector companies, which share properties with healthcare in focusing on direct contact with customers/patients. Malmbrandt and Åhlström’s development and validation process were both theoretical and empirical driven using a structured literature search, interviews with expert practitioners and workshops with researchers, academics and Lean expert practitioners. The instrument consists of 28 items measuring Lean adoption, each item with five response alternatives ranging from low Lean maturity to high Lean maturity. On the basis of reactions by their informants, Malmbrandt and Åhlstöm [28] deemed the content validity of the instrument to be satisfying, and they state that the instrument is sufficiently sensitive to detect changes over time. The aim of the present study was, based on Liker’s description of Lean, to further develop Malmbrandt and Åhlström’s instrument, which uses measures of staff perceptions of Lean maturity in a healthcare context. An additional aim was to describe and test the resulting instrument’s face validity, construct validity, internal consistency and stability. Permission to further develop Malmbrandt and Åhlström’s instrument for the healthcare sector was obtained from the authors.

## Method

**Fig. 2:**
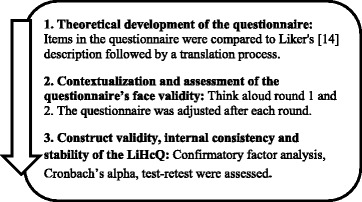
The stepwise process used in the study. The qualitative process is described in Step 1 and 2, and the quantitative process in Step 3

The development and evaluation process was based on a cross-sectional design with a mixed-method approach [32], comprising one theoretical step, followed by two steps based on the empirical data (Fig. 2). The study was approved by the Regional Ethical Review Board in Uppsala (Reg. no. 2014/525).

### Theoretical development of the questionnaire

Given our decision to base our questionnaire on Liker’s description of Lean, we first used a deductive approach to examine whether Malmbrandt and Åhlstöm’s instrument addressed all principles of Lean as described by Liker [14]; see Additional file 1. We found that three principles were not addressed, i.e. principles 8, 11 and 13 (cf. Fig. 1 for Liker’s description of Lean). Therefore, new items were developed to cover these principles. The next step was to translate the questionnaire from English to Swedish, which was done by the first author, and a back translation was subsequently carried out by a bilingual professional translator. Discrepancies between the versions were discussed and accounted for by our research group in collaboration with the translator [33, 34]. The resulting questionnaire prototype was called Lean in Healthcare Questionnaire (LiHcQ).

### Contextualization and assessment of the questionnaires face validity

To contextualize and validate the prototype of LiHcQ, the cognitive method Think Aloud (TA) was used to explore how healthcare staff perceived and interpreted the LiHcQ [35]. A convenience sample of seven units from different regions, different healthcare settings, hospital and primary care was obtained. First-line managers at the recruited units were instructed by their manager to ask staff with different professions, sex and age about their interest in participating. All participants in the TA had experience of Lean. A purposive sample of 12 staff with different professions (nurses, managers, physicians, physiotherapists, administrators/secretaries), sex and age participated in this step; the number of participants selected was based on suggestions made by Beatty and Willis [35]. Three participants worked in hospital and nine in primary care; both public non-profit and private for-profit providers were represented. Eleven were women, mean age 46 years (SD 10), and the most common profession was registered nurses; mean years worked at the present unit was 10 (SD 9) and mean years worked in the profession was 16 (SD 13).

The TA interviews were held by the first author in a private room at the participant’s respective workplaces during January and February 2015. Prior to the TA interviews, and again in connection with them, the participants received both written and verbal information about the study. At the beginning of the TA interview, participants were instructed to “think aloud” while they read the items in the LiHcQ prototype [36]. An initial sample of seven staff participated in the first rounds of TA sessions; based on their comments, the text in the LiHcQ was adjusted, and a new TA session with five other participants was conducted. Whenever the participant hesitated or reacted in any way while reading the LiHcQ, the researcher intervened, asking questions such as “I can see that you reacted to the statement, what are your thoughts about it?” [35]. The TA interview was completed by asking the participant to give his/her overall opinion about the questionnaire. The TA interview was terminated when no additional new information was obtained [36]. The interviews were audiotaped and transcribed verbatim [35].

The data were analyzed deductively, following Tourangeau’s [37] approach to TA data analysis. Thus, responses and comments were organized into four categories: comprehension, retrieval, judgment and response. According to Tourangeau, the category *comprehension* concerns whether words and phrases are difficult or impossible to understand; *retrieval* concerns whether responding is difficult because the needed information is not available; *judgment* concerns whether it is difficult to put information together to make a judgment and thereafter respond; *response* concerns difficulties in selecting a response option, e.g. if a participant hesitates to select between two response alternatives and would like to give an intermediate answer. This deductive analysis was conducted after both TA rounds. Adjustments to the questionnaire based on the analyses were made by the first author and discussed among all authors until consensus was reached. The adjusted version of the LiHcQ was thereafter tested for construct validity, internal consistency and stability.

### Construct validity, internal consistency and stability of the LiHcQ questionnaire

In this step, we recruited a convenience sample of staff working in public non-profit or private for-profit primary care; the primary care sector was selected due to the lack of research on Lean in this sector [10, 38]. All 52 primary care units, both public non-profit and private for-profit, in one region in central Sweden were asked to participate; 42 of the units wished to participate. Additionally, to increase the participation of private for-profit units, all 85 primary care units in one of the largest private for-profit healthcare providers in Sweden were asked to participate; six units agreed to participate. Included were units in primary care, with the exception of specialized units; those excluded focused on dermatology, nutrition, administration, or they were units with inpatients or call centers with telenurses. To be included, the units should have implemented Lean to some degree. Concerning the participant’s inclusion criteria, staff should have worked at least three months at their unit prior to data collection. The first-line manager at the units provided information about the study at their regular meetings, and all staff received written information from the researchers together with the questionnaire. The staff was also informed in writing that their consent to participate in the study would be given by their responding to the questionnaire. The adjusted and contextualized LiHcQ developed through the TA interview process was sent out in spring 2015, and 1040 staff members were eligible for inclusion. It was embedded in a larger questionnaire that also included items on, for instance, job satisfaction, general health and satisfaction with the care provided (data not presented here). During this phase, the LiHcQ was web-based, but those not responding on the web were sent a paper version. Two reminders were sent out. The response rate was 46% (481 of 1040). Of the 481 respondents, 386 had answered at least 50% or more of the LiHcQ items; further analyses used the data from these 386 respondents. An analysis of the non-respondents showed no significant difference between them and participants in sample concerning age, sex, years worked at the present unit and years worked in the profession, which indicates that the answers are representative. Most participants were female (*n* = 333), with a mean age of 50 years (SD 10); the most common profession was registered nurse (*n* = 150), and the mean number of years worked at the present healthcare unit was 9 (SD 9) (see Table 1 for sample characteristics). When testing construct validity, a confirmatory factor analysis (CFA) was employed on data from participants with complete data in all LiHcQ items (*n* = 243); using only complete data is common when conducting a CFA [39].

**Table 1:** Sample characteristics

	Responders to LiHcQ	Non-responders to LiHcQ	Subsample for analysis of test-retest reliability
Total number of participants, n	386	95	43
Participants at public non-profit//private for-profit provider healthcare units, n	320//66	71//24	36//7
Women//Men, n	333//49	85//8	35//8
Age;			
Md (Q_1−_Q_3_)	51 (43–58)	55 (47–59)	53 (41–57)
Mean (SD)	50 (10)	52 (10)	48 (11)
Profession, n			
- Nurses	150	31	20
- Licensed Practical Nurse (LPN)	25	18	2
- Manager	24	1	1
- Physiotherapist	41	3	8
- Occupational therapist	12	6	1
- Physician	66	4	5
- Administrator/secretary	39	24	1
- Dietician	2	1	2
- Social welfare officer/psychologist	35	6	2
Years worked at the present unit;			
Md (Q_1−_Q_3_)	5 (2–13)	7 (2–20)	5 (2–12)
Mean (SD)	9 (9)	12 (11)	7 (7)
Years worked in the profession;			
Md (Q_1−_Q_3_)	20 (10–30)	26 (15–35)	20 (11–28)
Mean (SD)	21 (12)	25 (12)	18 (11)

Participants in the validity and reliability analysis of the Lean in Healthcare Questionnaire (LiHcQ), as well as for non-responders, i.e. responders with missing answers to more than 50% of the LiHcQ items. *Md* Median, *Q* quartiles, *SD* standard deviation. When numbers do not add up to 386, 95 and 43, respectively, concerning professions this is because some participants have multiple functions

The data were analyzed using IBM SPSS Statistics, version 22. To investigate the construct validity of the LiHcQ, a CFA was performed using AMOS. CFA requires, as a rule of thumb, ten participants per variable [40]. The LiHcQ comprised 16 variables and, thus, the number of participants was sufficient. Among a large array of parameters describing goodness-of-fit, we selected the Chi-square test, the Root Mean Square Error of Approximation (RMSEA), the Comparative Fit index (CFI) and the Standardized Root Mean square Residual (SRMR), as recommended by Kline [40]. Kääriäinen [39] organizes goodness-of-fit metrics in two groups: absolute parameters and relative parameters. Chi-square and RMSEA are called *absolute parameters*, which indicate how well the hypothetical relationships between the variables match the observed relationships, i.e., how the model fits compared to no model at all. The Chi-square goodness-of-fit indicates that the model is acceptable when the relative Chi-square (Chi^2^/d.f.) is less than 3 and the p-value is larger than 0.05. However, the test has been criticized and other tests have been developed. RMSEA is one of them, and values for RMSEA below 0.08 may be considered acceptable [39]. In addition, Kline [40] recommends SRMR, i.e. the difference between the residuals in the covariance matrix of the employed sample and a hypothesized model. A good model has values less than 0.5 (theoretical range for values 0 to 1) [40]. *Relative parameters* test the adequacy of a theoretical model by comparing the sample covariance matrix to a null model where all variables are uncorrelated. One of the most common relative parameters is CFI, which we included in our study. A good fit is suggested if the value is greater than 0.90 [39]. We assessed internal consistency using Cronbach’s alpha coefficient, where values larger than 0.70 show acceptable performance. Stability in terms of test-retest reliability was evaluated through intra-class correlation coefficients (ICC) with 95% confidence intervals (CI). According to Cicchetti [41], ICC values can be considered poor if <0.40, fair when between 0.40 and 0.59, good between 0.60 and 0.74 and excellent if the value is ≥ 0.75. *P*-values less than 0.05 (two-tailed) were considered to indicate statistically significant results.

## Results

### Contextual adjustments and face validity of the preliminary questionnaire

The qualitative analyses of data from the first round of TA revealed that comments mostly concerned the category *comprehension*. The TA participants commented that some of the words employed did not fit into a healthcare context or that they had difficulties understanding certain words and phrases. Words and phrases that needed to be contextualized included e.g.: enabler, innovative, expert practitioner, standardized, infra-structural factors, to create flow in the processes, to level out the workload, and proactive planning. In the category *retrieval* there were no comments; in the categories *judgment* and *response* there were comments regarding a few items. Comments on the questionnaire as a whole concerned the opinion that it was too comprehensive and time consuming, and some participants mentioned that duplicate items seemed to occur.

One participant in the first TA round expressed the need for contextualizing the questionnaire: “*It feels like difficult language that I don*’*t really understand. And also it feels like a literal translation from English*, *a little stilted and strange*, …”

Another participant in the first round expressed the need for a shorter and contextualized questionnaire, however the participant stated that the questionnaire was relevant: “*It*’*s comprehensive and sort of difficult to respond to sometimes*, *to think about care and not factory production on some of them. I thought others were very good*.”

Adjustments after the first TA round mainly focused on changing the identified problematic words and phrases to everyday language in order to contextualize the questionnaire to the healthcare sector. The adjusted 31-item questionnaire was thereafter used in a second round of TA interviews. Comments concerning *comprehension* were now found to a much less extent, but a few words and phrases still needed attention. Regarding *judgment*, the participants expressed the need for additional information or clarification for some items. Comments concerning *retrieval* and *response* were few. Both TA rounds revealed that it was common for participants to fail to read or notice the information given on how to respond. Thus, the participants requested information that was, in fact, available in the written instructions, or they needed to read the information text repeatedly. Participants also expressed their lack of familiarity with maturity levels and statements. Another opinion expressed by most of the participants was, as in the first TA round, the need to reduce the number of items.

One participant in the second round expressed an overall feeling about the 31-item questionnaire; “*It feels a bit long. It can be hard to maintain your focus on each question all the way through. But otherwise there*’*s a lot that makes you think*, *we should deal with this or I*’*d like to do that*, *or be there. Lots of feelings like that*, *a lot*, *we have a long way to go*.”

Like after the first round, adjustments after the second TA round focused on re-phrasing some sentences using everyday words, to contextualize the questionnaire to the healthcare sector, and on writing clearer instructions. Mostly we chose words the participants themselves used in their context, expressed during the TA interviews. After the second TA round, the number of items in the LiHcQ was reduced based on both the theoretical framework by Liker and information given by several respondents in both TA rounds. A common statement from the participants was that the instrument was too comprehensive; they wondered who would have time to complete it. In this reduction process, we decided to retain at least one item for each of the 14 Liker principles. The *philosophy* domain is represented by only one principle in Liker’s description (see Fig. 1). However, to allow for better statistical assessments, three items were retained to represent this domain. In this process, 15 items were removed, and the resulting LiHcQ, shown in Additional file 2 (in English) and Additional file 3 (in Swedish), contains 16 items with five statements constructed as a maturity scale for each item.

### Testing the construct validity, internal consistency and stability of the questionnaire

Table 2 presents descriptive data for the items and the factors in the LiHcQ, including results on internal consistency and test-retest reliability. Internal missing values for the items varied from 0.7 to 17%, with two items having 10% or more missing answers (Table 2). Mean values for each item ranged from 1.6 to 3.5.

**Table 2:** Descriptive data for LiHcQ, internal consistency and test-retest

	*n* = 386			test –retest *n* = 43
*Factors* Item no in LiHcQ). (*Liker*’*s principle*)	Missing n (%)	Mean (SD)	Md (Q_1−_ Q_3_)	ICC (95% CI)
*Philosophy* α = 0.75				0.80 (0.63;0.89)
1). Employees participation in Lean (*Long*-*term thinking. Plan ahead and do investments even if they costs more at present*)	17 (4)	3 (1)	3 (2–4)	0.75 (0.53;0.86)
2). Ward manager participation in Lean (*Long*-*term thinking. Plan ahead and do investments even if they costs more at present*)	67 (17)	3 (1)	3 (3–4)	0.79 (0.59;0.89)
3). Allocated time for continuous improvements (*Long*-*term thinking. Plan ahead and do investments even if they costs more at present*)	6 (1)	3 (1)	2 (2–3)	0.64 (0.33;0.81)
*Processes* α = 0.86				0.77 (0.57;0.87)
6). Value stream mapping (*Create flow in the processes which makes problem visible*)	34 (9)	3 (1)	3 (2–3)	0.50 (0.06;0.73)
7). Standardization (*Have standardized work to achieve flow and continuous improvements. Encourage employee involvement*)	9 (2)	4 (1)	4 (3–4)	0.76 (0.56;0.87)
8). Plan with the patient in focus (*Level out the workload*)	18 (5)	3 (1)	3 (2–4)	0.55 (0.16;0.76)
9). Automatically quality controls (*Good quality from the beginning is achieved by teaching everyone to stop the process if quality problem occurs*)	39 (10)	3 (1)	3 (1–3)	0.65 (0.33;0.81)
10). Patient need control the work flow (*Avoid overproduction by producing only on customer demand*)	24 (6)	3 (1)	3 (2–4)	0.37 (0.19;0.66)
11). Visual improvements to guide the employees (*Use visualized signs in the process*, *to reduce errors*)	21 (6)	3 (1)	3 (2–4)	0.80 (0.62;0.89)
15). Technique and involve employees (*Use only techniques that are reliable*; *it shall support the employee and the processes*)	8 (2)	3 (1)	3 (3–4)	0.67 (0.38;0.82)
*People and partners* α = 0.60				0.88 (0.77;0.93)
4). A person who support Lean adoption at the unit (*Develop leaders from the organization that know the processes*, *know and can spread the Lean philosophy*)	21 (5)	2 (1)	1 (1–2)	0.94 (0.88;0.97)
5). Quality of given care (*Develop a culture where everyone share the organizations core values and want to improve the organization*)	17 (4)	3 (1)	4 (2–4)	0.75 (0.55;0.87
16). Employee collaboration with partners and suppliers (*Show respect to partners and suppliers and set up challenging goals for theme and help them to achieve it*)	14 (4)	3 (1)	2 (2–3)	0.66 (0.37;0.82)
*Problem*-*solving* α = 0.81				0.79 (0.61;0.89)
12). Evaluate each work task (*Be a learning organization through reflections and continuous improvements*)	20 (5)	3 (1)	2 (1–4)	0.80 (0.64;0.89)
13). Problem-solving (*To develop processes and to solve problems*; *go and see for yourself instead of trusting secondary information*)	14 (4)	3 (1)	3 (2–4)	0.68 (0.39;0.83)
14). Participation in decisions (*Decision making is a slow process and solutions are made in consensus*)	8 (2)	3 (1)	3 (3–4)	0.72 (0.47;0.85)

^α^Cronbach’s alpha, *LiHcQ* lean in healthcare questionnaire, *SD* standard deviation, *Md* median, *Q* quartiles, *ICC* intra-class correlation coefficient, *CI* confidence interval

**Fig. 3:**
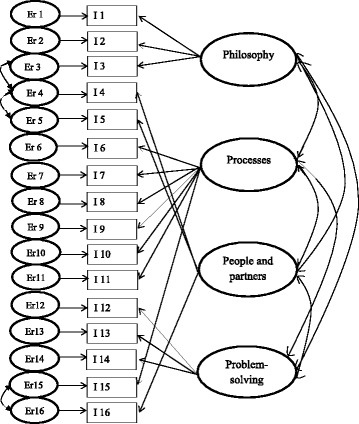
The factor structure model. The 4 domains to the right and the 16 items along with the error terms to the left. The arrows show the relationships

To test the construct validity of the LiHcQ and its correspondence with a model based on Liker’s 4P, a CFA was conducted on data from 243 respondents. The Chi-square test showed significance (*x* ^*2*^ = 221,625, *d.f*. = 95, *p* < 0.001), which is not desirable in this case; however, the other fit indices showed an acceptable model fit: the relative Chi-square was 2.33, RMSEA 0.07 (90%CI 0.06 to 0.09), SRMR 0.048 and CFI 0.93. The modification index suggested correlations between Item 3 and 4, as well as between Item 4 and 5. Item 3 belong to the factor *philosophy*, 4 and 5 to *people and partners*. The model also revealed a correlation between Item 15 and 16, Item 15 belonging to *processes* and 16 to *people and partners*. Correlations between the latent variables and the error terms for the above mentioned items were allowed in our model (see Fig. 3).

The internal consistency, measured using the Cronbach’s alpha coefficient for the total questionnaire, was 0.93 (*philosophy* 0.75, *processes* 0.86, *people and partners* 0.60, *problem*-*solving* 0.81) (Table 2). Stability, measured using ICC, showed acceptable values for all four factors; *philosophy* 0.80; *processes* 0.77; *people and partners* 0.88 and *problem*-*solving* 0.79.

## Discussion

Using a stepwise procedure, we developed a questionnaire – the LiHcQ – that measures staff perceptions of Lean adoption in the healthcare sector, based on Liker’s description of Lean. Validity and reliability, measured as face validity, construct validity, internal consistency and test-retest reliability, were acceptable on the whole.

### The theoretical development of the questionnaire

Recent reviews [3, 10, 38, 42] of Lean approaches in the healthcare sector have provided no clear candidate to use as a theoretical foundation when developing a questionnaire; they have largely focused on describing applied tools and techniques. Other descriptions of Lean were offered by Liker [14], Womack, Jones and Roos [15] and Shah and Ward [17]. Womack, Jones and Roos have received criticism for the lack of focus on people and partners in their description [16], and their framework was therefore excluded. Shah and Ward’s [17] view of Lean lacks decentralized decision-making and a long-term perspective, both of which are relevant to healthcare. Having respect for people and focusing on enabling their development is a central aspect in the theory of person-centered care [19, 43], which is emphasized in healthcare [21, 44]. These aspects, respecting and enabling people, are also included in Liker’s description of Lean and, therefore, constitute essential reasons for selecting his description of Lean as a basis for our instrument, despite the fact that Liker’s [14] description originated in the automobile industry.

The results show that Liker’s framework is generic enough to be used when adapting Malmbrandt and Åhlström’s [28] instrument in the context of healthcare. The participants’ responses show that Lean, as described by Liker, can be understood by staff in healthcare and it is already being in use.

### The contextual adjustments and face validity of the questionnaire

The qualitative method of TA interviews gave useful results in terms of contextualizing and validating the questionnaire for use in healthcare. When adjusting the questionnaire, words and phrases suggested by the participants were used. The strength of this procedure was that the participants came from different regions, different healthcare settings and had different professions. These variations reduce the risk of employing words and phrases in the LiHcQ that will only be understood by a limited group of healthcare staff. When reducing the size of the questionnaire, theoretical reasoning and empirical data from the TA were used to determine which items to discard, as recommended by Hox [45]. After finalizing the shorter version, the LiHcQ still represented all of Liker’s 14 principles [14] in form of a 16-item questionnaire with response alternatives as statements. The statements are constructed as a maturity scale influenced by the capability maturity model used in earlier studies of both Lean [24, 27] and in other areas [46]. One advantage of the LiHcQ is that it consists of only 16 items and takes approximately 15 min to complete, compared with Roszell’s [29] 110-item questionnaire. A common factor that affects response rate is the size of the questionnaire [47]. Conducting two rounds of TA interviews with different participants was another strength, as this procedure gave information on whether or not the initial adjustments were satisfactory. Previous studies have often failed to present the number of rounds that have been performed [48–50]. One difficult part of this process is to know when to terminate the TA. We conducted, as suggested, a total of twelve interviews and terminated when new insights ceased to emerge from the interviews [35].

### Construct validity, internal consistency and stability of the questionnaire

The construct validity of the LiHcQ, based on goodness-of-fit indices, was generally acceptable, and similar to values observed by Shah and Ward [17], who developed an instrument to measure Lean in industry. When conducting the CFA, we allowed the latent variables and error terms for some items in the model to correlate (Fig. 3). Correlations were allowed between error terms for Item 3 and 4. Item 3 belongs to the factor *philosophy* and focused if time for continuously improvements is approved, Item 4 belongs to *people and partners*; if a specific person is designated to encourage and support staff adopting Lean. The similarity between Item 3 and 4, which theoretically justifies the association, is that both items focus on to what extent the organization allocates time and resources to Lean. The correlation between Item 4 and 5, both in the factor *people and partners*, can be explained by the mutual focus on showing respect to the staff by involving them in Lean adoption and letting them grow through challenges. The model also showed a correlation between Item 15, belonging to *processes*, and Item 16, to *people and partners*, the similarity being that both items concern whether the staff are trusted and able to participate in or make decisions. The difference between them is that the focus of Item 15 is on improving the processes, while Item 16 primarily concerns staff having relations based on showing respect for partners and suppliers, the aim being to enable all involved to grow.

The internal consistency assessed by Cronbach’s alpha showed acceptable values for three factors (*philosophy*, *processes* and *problem*-*solving*), while the α-value for *people and partners* was 0.60. Items in the factor *people and partners* are 4, 5 and 16. The low α-value can be explained by the low dispersion in responses to Item 4 (Table 2). ICCs showed an acceptable stability for all factors. Some participants have not responded to some items, which can be explained by lexical problems; certain words are familiar or have a meaning for one group, but not for others [51]. However, results from the TA interviews show that the LiHcQ was not generally difficult to understand. Another reason for missing values may be that the LiHcQ was placed at the end of a longer questionnaire with a total of 77 items, which could have lessened participants’ enthusiasm for completing the LiHcQ. The items with most missing values were 2 (17%) and 9 (10%) (see Table 2). Item 2 concerned the first-line manager’s commitment to Lean. One reason for not responding to this Item could be that participants felt they did not have firsthand information about their first-line manager’s opinions about Lean. Item 9 concerned the extent to which the healthcare unit had automatic quality controls. When conducting the TA interviews, some participants expressed that they or their colleagues e.g. secretaries, worked more isolated from the rest. This could also explain some of the missing data. However, according to Liker [14], the whole unit should have knowledge about what aspects of Lean are being adopted.

In the present study, we used a convenience sample for testing the construct validity, internal consistency and stability of the LiHcQ questionnaire, which limits the generalizability of the findings. When recruiting primary care units, only 6 of 85 units from one of the largest private for-profit healthcare providers in Sweden wished to participate. The reason for this has not been analyzed. However, non-participation could be steered by that the units did not consider themselves to have adopted Lean or that they feel they have only adopted parts of Lean mixed with other improvement strategies. We did ask for units that had implemented Lean to some degree. Another reason could be that the healthcare staff are strained and need to reduce the number of extra commitments. Another important factor impacting the result is the low response rates, and missing data in the LiHcQ which may indicate possible non-response bias [33]. When conducting a CFA it is recommended to use cases with complete data on all items [39], consequently the number of cases in this study decreased. However, no differences were found between responders and not responders. Analyses of the 481 responders and non-responders regarding age, sex, years worked at the present unit and years worked in the profession showed no significant differences between the groups, indicating that the results are not biased as regards these factors. The fact that nursing was the profession most represented in the study is also a factor that limits the generalizability. On the other hand, nurses are the largest licensed group in the healthcare sector [52, 53]. Strength in the study is that the staff varied in terms of profession (nurses, managers, physicians, physiotherapists, administrators/secretaries, Licensed Practical Nurses (LPNs), dieticians, social welfare officers, psychologists and occupational therapists), age, geographic location, unit size and public non-profit vs. private for-profit providers [54].

## Conclusions

The current paper presents a questionnaire that measures staff perceptions of Lean adoption in the healthcare sector, based on Liker’s principles of Lean. It describes the stepwise development of the questionnaire and its psychometric properties. They were generally acceptable, which suggests that the questionnaire can be used in the healthcare sector as intended. We suggest that future research focus on verifying the usability of the questionnaire in other healthcare settings and on adjusting the instrument if needed.

## Additional files

Additional file 1: Malmbrandt and Åhlstöm’s (2013) instrument and the LiHcQ divided into Liker’s (2004) principles and domains. (DOCX 17 kb)

Additional file 2: The Lean in Healthcare Questionnaire (LiHcQ) (in English). (DOCX 59 kb)

Additional file 3: The Lean in Healthcare Questionnaire (LiHcQ) (in Swedish). (DOCX 30 kb)

## Abbreviations

4P: Philosophy, processes, people and partners and problem-solving
α: Cronbach’s alpha
CFA: Confirmatory factor analysis
CFI: Comparative fit index
CI: Confidence interval
Er: Error term
I: Item
ICC: Intra-class correlation coefficient
LiHcQ: Lean in healthcare questionnaire
Md: Median
RMSEA: Root mean square error of approximation
SD: Standard deviation
SRMR: Standardized root mean square residual
TA: Think aloud
