# Supplementary material for: A questionnaire measuring staff perceptions of Lean adoption in healthcare: development and psychometric testing
Source: BMC Health Serv Res. 2017 Mar 24;17:235. doi: 10.1186/s12913-017-2163-x (PMC5364711; doi:10.1186/s12913-017-2163-x)
Supplement: Supplementary file 1 — Malmbrandt and Åhlstöm’s (2013) instrument and the LiHcQ divided into Liker’s (2004) principles and domains. (DOCX 17 kb) [file 12913_2017_2163_MOESM1_ESM.docx]

**Additional file 1** Malmbrandt and Åhlstöm’s [28] instrument and the Lean in Healthcare Questionnaire (LiHcQ) divided into Liker’s [14] principles and domains.

Items kept in the **LiHcQ** are in boldface text. Items marked * are newly developed for the present study.

| Items by Malmbrandt and Åhlstöm [28] | Items in the **LiHcQ** | Liker’s principles | Liker’s four domains (4P) |
| --- | --- | --- | --- |
| Employee commitment  Employee understanding  Management commitment  Management understanding  Time for improvement work | **(1) Employee commitment**  **(2) Management commitment**  **(3) Time for improvement work** | 1  1  1  1  1 | Philosophy  -long-term thinking |
| Value stream mapping  Workplace design for flow  Connecting the processes  Bi-directional information flow | **(6) Value stream mapping** | 2  2  2  2 | Processes  -eliminate waste |
| Pull system | **(10) Pull system** | 3 |  |
| Proactive planning | **(8) Proactive planning** | 4 |  |
| Built-in quality | **(9) Built-in quality** | 5 |  |
| Standardized tasks  Formalization of work standards | **(7) Standardized tasks** | 6  6 |  |
| Visual signals  Visualization of information  Visualization of improvements | **(11) Visualization of improvements** | 7  7  7 |  |
| - | **(15) Use reliable technique that supports employees and processes*** | 8 |  |
| Change agent | **(4) Change agent** | 9 | People and partners  -respect, challenge and grow them |
| Identification of customer value  Employee training  Multifunctional teams | **(5) Identification of customer value** | 10  10  10 |  |
| - | **(16) Show respect for partners and suppliers*** | 11 |  |
| Structured problem solving | **(13) Structured problem-solving** | 12 | Problem-solving  -continuous improvements |
| - | **(14) Make decisions slowly and by consensus*** | 13 |  |
| Employees measure and follow up work  Resources for improvement work  Employee participation in improvement work  Focus of improvement work  Customer involvement  Sustaining improvements | **(12) Employees measure and follow up work** | 14  14  14  14  14  14 |  |
