# Supplementary material for: A questionnaire measuring staff perceptions of Lean adoption in healthcare: development and psychometric testing
Source: BMC Health Serv Res. 2017 Mar 24;17:235. doi: 10.1186/s12913-017-2163-x (PMC5364711; doi:10.1186/s12913-017-2163-x)
Supplement: Supplementary file 3 — The Lean in Healthcare Questionnaire (LiHcQ) (in Swedish). (DOCX 30 kb) [file 12913_2017_2163_MOESM3_ESM.docx]

**Additional file 3: The Lean in Healthcare Questionnaire (LiHcQ)**

**Nedanstående frågor syftar till att ge en beskrivning av om och i vilken utsträckning Lean förekommer på din arbetsplats.**

Du besvarar enkäten genom att **markera det svarsalternativ som bäst stämmer in på din arbetsplats**. Påstående 1 innebär att det inte finns något av det efterfrågade på vårdenheten; påstående 2-5 innebär att det efterfrågade finns på din arbetsplats och att nivån ökar gradvis. **Du tar först ställning till påstående 1, stämmer inte det in på din arbetsplats behöver du ta ställning till påstående 2 osv.** **Om du t.ex. anser att påstående 4 stämmer in på din arbetsplats ska även påståendena 2 och 3 göra det,** för att svara markerar du endast påstående 4 på den frågan.

**1. Medarbetarnas engagemang i Lean**

(Ord med en * finns förklarade efter frågan. Alla beskrivningar som ges nedan har samma betydelse i hela denna del av enkäten. Då ordet återkommer i en annan fråga kan du gå tillbaka till den tidigare beskrivningen).

| ( ) 1. | Medarbetarna vid vårdenheten visar inget engagemang alls eller är negativt inställda till Lean*. |
| --- | --- |
| ( ) 2. | Medarbetarna ser Lean som ett tillfälligt projekt och är bara beredda att lägga begränsad tid och engagemang på förbättringsarbete**. |
| ( ) 3. | Medarbetarna stödjer Lean. De lägger tid och engagemang på att komma på förbättringsidéer men inte på att omsätta idéerna till nya sätt att arbeta på eller för att lösa problem. |
| ( ) 4. | Medarbetarna är drivande för att Lean ska bli en del av det dagliga arbetet. De vill utveckla arbetet utifrån förbättringsidéer och har långsiktiga lösningar på problem. |
| ( ) 5. | Alla vet att medarbetarnas insatser är betydelsefulla i Lean. Förbättringsarbete anses vara en viktig del i det dagliga arbetet där fokus ligger på både nya lösningar och på att bibehålla tidigare lösningar. |

** Vanligt förekommande inom Lean är att medarbetarna fokuserar på att ständigt förbättra. Flöden innebär att vården ges i en sammanhållen kedja utan avbrott. Vårdkvalitet och vad patienten efterfrågar är i fokus.

**Förbättringsarbete innebär att medarbetarna vid vårdenheten löser problem och förbättrar t.ex. sina arbetssätt, vårdkedjor, flöden och vårdkvalitet. Att utvärdera arbetssätt och genomförda förbättringar ingår alltid i förbättringsarbete.*

**2. Vårdenhetschefens engagemang i Lean,** är du vårdenhetschef skattar du dig själv som chef

| ( ) 1. | Vårdenhetschefen visar inget engagemang alls eller är negativt inställd till Lean. |
| --- | --- |
| ( ) 2. | Vårdenhetschefen ser Lean som ett tillfälligt projekt och är bara beredd att lägga  begränsad tid och resurser på förbättringsarbete. |
| ( ) 3. | Vårdenhetschefen stödjer Lean och godkänner att tid och resurser läggs på förbättringsarbete. Han/hon tar dock ingen aktiv roll för att Lean ska bli en del av det dagliga arbetet utan ser det huvudsakligen som något medarbetarna ska göra. |
| ( ) 4. | Vårdenhetschefen ställer frågor på ett ”coachande” sätt om problem och förbättringsförslag och är drivande för att Lean ska bli en del av det dagliga arbetet. |
| ( ) 5. | Alla anser att vårdenhetschefen har stor betydelse i Lean. Vårdenhetschefens ”coachande” sätt har lett till att medarbetarna har kommit på nya lösningar som förbättrar för dem och/eller patienterna. Det är lika stort fokus på nya lösningar som på att bibehålla tidigare. |

**3. Tid för förbättringsarbete**

| ( ) 1. | Ingen tid är specifikt avsatt för förbättringsarbete. |
| --- | --- |
| ( ) 2. | I sällsynta fall avsätts viss tid för förbättringsarbete. |
| ( ) 3. | De flesta team* på vårdenheten har frekventa förbättringsmöten eller liknande. Olika team har kommit olika lång rörande hur förbättringsarbete bedrivs. |
| ( ) 4. | Alla team vid vårdenheten har frekventa förbättringsmöten eller liknande vilket innebär att alla medarbetare är involverade. |
| ( ) 5. | Förbättringsarbetet är en del av det dagliga arbetet och alla anser att förbättringsarbete har stor betydelse för vårdens kvalitet. |

**Ett team består av medarbetare med olika funktioner, det kan vara två eller flera personer från olika yrkeskategorier, men även medarbetare från samma yrkeskategori kan utgöra ett team med olika funktioner om de bidrar med olika kunskaper.*

**4. Stödjande resurs för Lean-arbetet**

| ( ) 1. | Det finns ingen person/flera personer utsedda lokalt på vårdenheten för att stödja medarbetarna i syfte att Lean ska blir en del av det dagliga arbetet. |
| --- | --- |
| ( ) 2. | En person/flera personer har utsetts lokalt på vårdenheten som ska stödja medarbetarna för att Lean ska blir en del av det dagliga arbetet. |
| ( ) 3. | Den person/de personer som ska stödja medarbetarna har fått den träning som behövs för uppgiften. |
| ( ) 4. | Den person/de personer som har utsetts arbetar nära medarbetarna och är en drivande kraft för förbättringsarbete. |
| ( ) 5. | Alla anser att den person/de personer som stödjer medarbetarna har en stor betydelse, har stor kompetens och ger ett bra stöd till teamen. |

**5. Vårdkvalitet**

| ( ) 1. | Ingen på vårdenheten försöker förstå vad patienterna värdesätter*. |
| --- | --- |
| ( ) 2. | Medarbetarna på vårdenheten har på ett ostrukturerat sätt börjat undersöka vad patienterna värdesätter. Teamen har kommit olika långt för att få fram denna information. |
| ( ) 3. | Vad patienterna värdesätter och vad som bidrar och inte bidrar till detta diskuteras aktivt i de flesta team. |
| ( ) 4. | De flesta kan se och beskriva vad som sker på vårdenheten och vad de personligen gör som skapar värde för patienterna och vad som inte skapar värde. |
| ( ) 5. | Alla på vårdenheten har ett nytänkande och är mycket skickliga i att identifiera vad patienterna värdesätter. Vad patienterna värdesätter utvärderas hela tiden. |

**Vad patienterna värdesätter är sådant som bidrar till ökad vårdkvalitet eller vad patienten efterfrågar. Vad patienterna inte värdesätter kan vara att vänta, inte ha möjlighet att påverka, att inte få information eller att personalen behöver leta material.*

**6. Att kartlägga vad patienterna värdesätter och inte värdesätter i vården**

| ( ) 1. | På vårdenheten kartläggs* inga vårdkedjor för att höja vårdkvaliten eller för att beskriva vad patienterna värdesätter respektive vad de inte värdesätter. |
| --- | --- |
| ( ) 2. | Några eller delar av vårdkedjorna på vårdenheten är kartlagda. Kartläggningarna varierar i kvalitet och kan vara gamla. Genom återkommande problem på vårdenheten har olika sorters slöseri** uppdagats. |
| ( ) 3. | Utifrån att medarbetarna vet vad patienterna värdesätter och vad som kan höja vårdkvaliten har medarbetarna upptäckt olika sorters slöseri. De vanligaste vårdkedjorna på vårdenheten är kartlagda, vissa är detaljerade medan andra är mer ytligt utförda. |
| ( ) 4. | På vårdenheten finns uppdaterade och detaljerade kartläggningar av vårdkedjorna.  Kartläggningarna uppdateras minst en gång per år och har placerats synliga utifrån var medarbetarna behöver informationen. |
| ( ) 5. | Vid vårdenheten finns en välutvecklad struktur för att genomföra och använda information från kartläggningar. Kartläggningarna uppdateras kontinuerligt och används för att förbättra vården. |

**Att kartlägga vårdkedjan innebär att medarbetarna i detalj kartlägger patientens vård, t.ex. dokumenteras hur länge patienten har direktkontakt med vårdpersonal, hur länge personal eller patient får vänta, vilka vårdinsatser som utförs och av vem och hur lång tid det tar. Syftet med att kartlägga flöden i vårdkedjor är att få information om vad som kan förbättras genom att minska slöseri och öka vårdkvalitén.*

***Slöseri kan vara väntetider för både personal och patienter, att patienterna inte får förståelig information eller att personalen letar material. Att upptäcka slöseri kan vara att medarbetarna har uppmärksammat att det alltid saknas material i förrådet. Att leta material eller finna annan tillfällig lösning är slöseri och grundorsaken till problemet är inte löst.*

**7. Att göra på samma sätt**

| ( ) 1. | På vårdenheten följs inga rutiner* då arbetsuppgifter utförs. |
| --- | --- |
| ( ) 2. | Några team på vårdenheten har börjat skapa och använda rutiner. Dessa är inte alltid nedskrivna eller kan vara i form av enkla checklistor. |
| ( ) 3. | Vissa team har rutiner. Rutinerna börjar bli tydligare, mer detaljerade och nedskrivna. |
| ( ) 4. | De flesta team har rutiner. Teamen har också börjat undersöka om valda rutiner alltid används. |
| ( ) 5. | Alla på vårdenheten är mycket skickliga på att utveckla och följa rutiner. När problem och fel uppdagas används rutinen för att finna orsaken till fel/problem. Frågor som kan ställas är t.ex. följdes rutinen, behöver den förtydligas eller helt revideras, behövs mer kunskap? |

**Med rutiner menas det arbetssätt som vårdenheten själv har bestämt att de ska följa. Det kan vara att kalibrering av apparatur sker vissa dagar eller att alla tar samma prover vid misstanke om en viss diagnos. Påståendena avser inte nationella riktlinjer, vårdhandboken, lokala riktlinjer eller liknande utan avser t.ex. de rutiner vårdenheten har skapat för att följa nationella riktlinjer.*

**8. Planera arbetet utifrån patienternas behov**

| ( ) 1. | På vårdenheten är förändringar i patienttillströmning till stor del oförutsedda liksom vilka  resurser (t.ex. tid, personer, kompetens) som krävs. Vårdenheten planerar inte i förväg*. |
| --- | --- |
| ( ) 2. | Medarbetarna på vårdenheten har börjat granska förändringar i patienttillströmningen och vilka resurser (t.ex. tid, personer, kompetens) som krävs. |
| ( ) 3. | Medarbetarna på vårdenheten planerar när olika resurser ska användas utifrån statistik om  patienttillströmning. Detta kan t.ex. innebära att flera arbetar i början av veckan eftersom flest patienter kommer då. |
| ( ) 4. | För att mer exakt kunna förutse vilka resurser som krävs har vårdenheten tidig patientkontakt. Det kan innebära att vårdenheten erbjuder patienterna en hälsokontroll. |
| ( ) 5. | Alla på vårdenheten har ett nytänkande och är mycket skickliga på att planera i förväg. För att få ett bra flöde i vårdkedjorna** används olika sätt för att engagera*** patienterna i sin vård och hälsa. |

** Att planera i förväg för att utjämna arbetsmängden innebär att vårdenheten försöker uppskatta patienternas behov i förväg. Det kan innebära att hänsyn tas till att fler patienter söker vård en viss dag eller att det under en period är många vaccinationer.*

*** Att ha bra flöden i vårdkedjorna innebär att vårdkedjan är sammanhållen och har rätt vårdkvalitet. Praktiskt innebär det att medarbetarna strävar efter att minska väntetider för patient och personal och att rätt medarbetare ger rätt vård vid rätt tidpunkt. Olika resurser t.ex. information eller material, ska finnas lättillgängliga och synliga för medarbetarna där de behövs. Ett exempel på en vårdkedja kan vara från att en medarbetare bokar patienten, patienten tas emot och registreras av kollega vid besöket, själva besöket o.s.v.*

****Att engagera patienterna för att få ett bra flöde i vårdkedjorna kan göras genom att t.ex. uppmana dem att ringa sjukvårdsrådgivningen i större utsträckning, förbereda vissa undersökningar/provtagningar hemma eller besvara frågor hemma inför besöket.*

**9. Att säkerställa vårdkvalitet genom automatiska kvalitetskontroller***

| ( ) 1. | Medarbetarna vid vårdenheten känner inte till hur kvalitetskontroller kan ske automatiskt eller så tar kvalitetskontroll upp mycket tid. Det kan även vara att medarbetarna har låg medvetenhet angående vårdkvalitet generellt. |
| --- | --- |
| ( ) 2. | Medarbetarna vid vårdenheten har börjat undersöka hur automatiska kvalitetskontroller kan ske för att kontrollera vårdkvaliten och arbetssätt. Tillvägagångssättet är dock ostrukturerat och inom ett fåtal team. |
| ( ) 3. | Några team har ett strukturerat tillvägagångssätt för att finna hur automatiska kvalitetskontroller kan införas och användas. |
| ( ) 4. | Nästan alla på vårdenheten är engagerade för att kvalitetskontroller ska ske automatiskt. För att det ska bli rätt från varje medarbetare förebygger de på olika sätt att fel uppstår och de granskar även kvalitén på sin egen arbetsuppgift. |
| ( ) 5. | Alla vid vårdenheten är mycket skickliga på både att införa och använda automatiska  kvalitetskontroller. Arbetsuppgifterna har utformats för att säkerställa att det blir rätt kvalitet från början och liten tid går till att kontrollera kvalitet. |

**Att ha automatiska kvalitetskontroller kan innebära att en viktig uppgift måste dokumenteras i journalen för att kunna fortsätta dokumentera. Det kan även vara att apparater inte går att använda om de används felaktigt eller kontakter och stickor som är utformade att bara passa på ett sätt.*

**10. Patienternas behov styr arbetet**

| ( ) 1. | På vårdenheten är det inte vad patienterna efterfrågar* som styr vårdinsatserna. |
| --- | --- |
| ( ) 2. | Teamen har börjat arbeta utifrån vad patienterna efterfrågar. Att utföra arbete som inte efterfrågats är fortfarande vanligt vilket kan vara att förbereda ett patientbesök eller undersökning långt i förväg eller utan ordination. |
| ( ) 3. | De flesta team arbetar utifrån vad patienterna efterfrågar och för att få ett bra flöde i vårdkedjorna använder teamen olika tecken och signaler**. Medarbetarna har börjat hjälpa varandra för att hela vårdenheten ska få bra flöden. |
| ( ) 4. | Alla team arbetar efter efterfrågan men har kommit olika långt. Att veta i förväg vad som efterfrågas underlättar bra flöden i vårdkedjorna. Medarbetarna vet vad som sker både före och efter sin del av arbetet och de använder tecken och signaler för att visa när nästa medarbetare ska utföra sin vårdinsats. Genomförda förbättringar bibehålls. |
| ( ) 5. | Medarbetarna vid vårdenheten har ett välutvecklat förhållningssätt till att arbeta utifrån efterfrågan. Det sker en ständig utveckling i att förbättra tecken och signaler för att få bra flöden i vårdkedjorna. |

**Att efterfrågan styr kan i praktiken innebära att fler personal arbetar då det är högt patienttryck, t.ex. en viss veckodag. Det kan även vara att man ändrat öppettiderna eftersom flera patienter efterfrågat det.*

***Synliga tecken eller signaler kan vara färgmarkeringar, text, ljud eller tecken i t.ex. datorn, på lappar eller på en magnettavla.*

*Tecken/signaler kan visa t.ex. att diktat är färdiga att signeras, att patienten har kommit till sin inbokade tid eller att patienten har träffat läkaren och att undersköterskan nu kan utföra sin del. Tecknen eller signalerna kan även visa t.ex. var material, apparatur eller personal finns eller att något håller på att ta slut eller vilka förbättringar, problem och avvikelser som finns.*

**11. Att synliggöra förbättringar för att alla ska kunna arbeta utifrån dessa**

| ( ) 1. | Vid vårdenheten synliggörs* inga genomförda förbättringar. |
| --- | --- |
| ( ) 2. | Inom delar av vårdenheten synliggörs vissa förbättringar men på ett ostrukturerat sätt. Medarbetarna vill lära sig och veta mer om hur förbättringar kan synliggöras. |
| ( ) 3. | Vid nästan hela vårdenheten synliggörs förbättringar och placeras utifrån medarbetarnas behov. Beskrivningarna av förbättringarna är aktuella och medarbetarna prövar sig fram gällande vad som ska synliggöras. |
| ( ) 4. | Vid hela vårdenheten synliggörs förbättringar och man placerar dessa utifrån medarbetarnas behov. Det som synliggörs är aktuellt och handlar om olika saker. |
| ( ) 5. | Alla vid vårdenheten har ett nytänkande och är mycket skickliga på att synliggöra förbättringar. Det som synliggörs är aktuellt och placeringen är väl genomtänkt. |

**Att synliggöra olika förbättringar kan vara att medarbetaren sätter upp foton på vad man har beslutat ska finnas på ett behandlingsrum eller att antal fel, avvikelser och åtgärder på vårdenheten finns synliga. Det kan också vara beskrivningar av nya arbetssätt, utvärderingar och vidareutvecklingar av arbetssätt eller hur medarbetarna löst problem från grunden.*

**12. Att utvärdera sitt arbete**

| ( ) 1. | Vårdenhetschefen eller högsta ledningen beslutar vanligtvis om utvärderingar* av vården och det är svårt för medarbetarna att påverka dess utformning och genomförande. |
| --- | --- |
| ( ) 2. | Några medarbetare har för egen del börjat söka efter olika metoder för att utvärdera sitt arbete och olika vårdkedjor. |
| ( ) 3. | Medarbetarna har själva börjat utvärdera vissa arbeten och vårdkedjor, vissa medarbetare har även utformat olika tillvägagångssätt för hur utvärdering ska gå till. |
| ( ) 4. | Utvärderingar utförs i olika stor utsträckning på vårdenheten. Utvärderingarna följer upp arbetet och de flesta vårdkedjorna och inte enskilda medarbetare. |
| ( ) 5. | Alla vid vårdenheten är mycket skickliga på att utvärdera arbetet och vårdkedjor. Medarbetarna både utvecklar och använder nya sätt att utvärdera genomförda förbättringar på. |

** Att utvärdera innebär att mäta och följa upp sitt arbete eller en vårdkedja, t.ex. ska ändrade öppettider eller ett nytt arbetssätt utvärderas för att beslut kan fattas* *om huruvida förändringar ska bestå/bli mer permanent. För att utvärdera kan medarbetarna t.ex. mäta patientgenomströmningen, hur nöjda patienterna är eller hur lång tid det tar innan patienten tillfrisknat.*

**13. Problemlösning**

| ( ) 1. | Förbättringsarbeten är oplanerade och ostrukturerade. Ofta fokuserar medarbetarna på själva problemet och inte på grundorsaken till problemet. Fel och problem löses genom ”brandkårsutryckningar”. |
| --- | --- |
| ( ) 2. | Förbättringsarbeten vid vårdenheten är mer strukturerat och medarbetarna har börjat använda olika metoder för att lösa problem på*. De söker efter grundorsaken till fel och problem. |
| ( ) 3. | Medarbetarna är skickliga på problemlösning, rutinmässigt löser de fel och problem på olika sätt. |
| ( ) 4. | Medarbetarna provar sig fram med förbättringsarbete. De utvärderar alltid genomförda förbättringar och justerar utifrån utvärderingens resultat. |
| ( ) 5. | Vid vårdenheten finns en välutvecklad struktur för förbättringsarbete. Förbättringar genomförs för att lösa problem och för att vidareutveckla flöden, vårdkedjor och arbetssätt. |

**Att använda olika metoder eller verktyg för problemlösning kan vara att medarbetarna undersöker grundorsaken till fel och problem, att de utvärdera sitt arbetssätt eller att berörda medarbetare själva ser och undersöker problem istället för att förlita sig på andrahandsinformation.*

**14. Delaktighet i beslut**

| ( ) 1. | Beslut fattas av vårdenhetschefen utan kommunikation med medarbetarna. |
| --- | --- |
| ( ) 2. | Ibland medverkar medarbetarna vid informationsträffar om viktiga beslut och förändringar men det är oftast vårdenhetschefen som fattar beslut utan dialog med medarbetarna. |
| ( ) 3. | Vårdenhetschefen och medarbetarna har strukturerade möten då beslut fattas, vårdenhetschefen är intresserad och tar ibland hänsyn till medarbetarnas åsikter. |
| ( ) 4. | Vid vårdenheten finns strukturerade möten och vårdenhetschefen strävar efter samförstånd. |
| ( ) 5. | Vid vårdenheten finns en välutvecklad struktur då beslut ska fattas. Medarbetarna är delaktiga, engagerade och beslut fattas i samförstånd. Olika alternativ övervägs alltid. |

**15. Teknik och medarbetarna**

| ( ) 1. | Medarbetarna involveras inte då produkter köps in. |
| --- | --- |
| ( ) 2. | Vårdenhetschefen har börjat rådgöra med berörda medarbetare vid inköp av produkter. |
| ( ) 3. | Några medarbetare involveras då produkter ska köpas in. Produkter som köps in ska förbättraflöde, höja vårdkvalitet eller stödja medarbetarna i sitt arbete. |
| ( ) 4. | Berörda medarbetare är alltid involverade och delaktiga när nya produkter ska köpas in. |
| ( ) 5. | Vid vårdenheten finns en välutvecklad struktur kring inköp och införandet av produkter. Produkterna testas och utvärderas alltid av berörda innan de införs. |

**16. Samarbetspartners och leverantörer**

| ( ) 1. | Medarbetarna har ingen kontakt med samarbetspartners* och leverantörer*. |
| --- | --- |
| ( ) 2. | Några på vårdenheten har kontakt med samarbetspartners och leverantörer rörande beställningar eller annat. |
| ( ) 3. | Några på vårdenheten har kontinuerlig kontakt med samarbetspartners och leverantörer och förhandlar med dem t.ex. rörande pris, leveranstider eller väntetider. |
| ( ) 4. | Flera på vårdenheten förhandlar med och stöttar samarbetspartners och leverantörer för att deras tjänster t.ex. ska passa de flöden som finns på vårdenheten, hålla hög kvalitet eller sänka leveranskostnader. |
| ( ) 5. | Vid vårdenheten finns välutvecklade samarbeten och en struktur rörande att förhandla med och stödja samarbetspartners och leverantörer. |

**Samarbetspartners och leverantörer kan vara Apoteket, andra vårdinstanser utanför den egna vårdenheten, inköpta tjänster såsom städ eller vaktmästeri, de som levererar förbrukningsartiklar eller kontorsmaterial.*
